# Does conventional morphological evaluation still play a role in predicting blastocyst formation?

**DOI:** 10.1186/s12958-022-00945-y

**Published:** 2022-04-19

**Authors:** Xiaoming Jiang, Jiali Cai, Lanlan Liu, Zhenfang Liu, Wenjie Wang, Jinhua Chen, Chao Yang, Jie Geng, Caihui Ma, Jianzhi Ren

**Affiliations:** 1 Reproductive Medicine Center, Xiamen University Affiliated Chenggong Hospital, Xiamen, 361003 Fujian China; 2 School of Medicine, Xiamen University, Xiamen, 361005 Fujian China

**Keywords:** Cell number, Symmetry, Fragmentation level, Early cleavage, Blastulation

## Abstract

**Background:**

Advanced models including time-lapse imaging and artificial intelligence technologies have been used to predict blastocyst formation. However, the conventional morphological evaluation of embryos is still widely used. The purpose of the present study was to evaluate the predictive power of conventional morphological evaluation regarding blastocyst formation.

**Methods:**

Retrospective evaluation of data from 15,613 patients receiving blastocyst culture from January 2013 through December 2020 in our institution were reviewed. Generalized estimating equations (GEE) were used to establish the morphology-based model. To estimate whether including more features regarding patient characteristics and cycle parameters improve the predicting power, we also establish models including 27 more features with either LASSO regression or XGbosst. The predicted number of blastocyst were associated with the observed number of the blastocyst and were used to predict the blastocyst transfer cancellation either in fresh or frozen cycles.

**Results:**

Based on early cleavage and routine observed morphological parameters (cell number, fragmentation, and symmetry), the GEE model predicted blastocyst formation with an AUC of 0.779(95%CI: 0.77–0.787) and an accuracy of 74.7%(95%CI: 73.9%-75.5%) in the validation set. LASSO regression model and XGboost model based on the combination of cycle characteristics and embryo morphology yielded similar predicting power with AUCs of 0.78(95%CI: 0.771–0.789) and 0.754(95%CI: 0.745–0.763), respectively. For per-cycle blastocyst yield, the predicted number of blastocysts using morphological parameters alone strongly correlated with observed blastocyst number (*r* = 0.897, *P* < 0.0001) and predicted blastocyst transfer cancel with an AUC of 0.926((95%CI: 0.911–0.94).

**Conclusion:**

The data suggested that routine morphology observation remained a feasible tool to support an informed decision regarding the day of transfer. However, models based on the combination of cycle characteristics and embryo morphology do not increase the predicting power significantly.

**Supplementary Information:**

The online version contains supplementary material available at 10.1186/s12958-022-00945-y.

## Introduction

Advances in embryo culture systems promote the currency of moving toward blastocyst transfer [1]. Extending the duration of embryo culture to the blastocyst stage may have several advantages, including a higher implantation rate over cleavage transfer and the potential to reduce the number of embryos transferred. Theoretically, blastocyst culture may help select the most viable embryo for transfer. However, low blastocyst formation rate may lead to an increase risk of transfer cancellation [1]. While the good-prognosis patients may benefit from blastocyst transfer, the patients with unfavorable characteristics, such as poor response or advanced age, may suffer an increased incidence of canceled transfers [2, 3]. Canceled transfer may add to the burden of infertile couples, both emotionally and economically. Therefore, predicting the possibility of blastocyst formation might be the key to giving a meaningful informed consent before providing blastocyst culture, especially for patients with few embryos available on day 3.

In efforts to facilitate the clinical decision-making before blastocyst culture and transfer, several models were developed to predict the blastocyst transfer cancellation or blastocyst formation for individual patients [4, 5], which demonstrated that the greatest predict value may lie in the number and quality of day 3 embryos. In recent years,‘OMICS’ technologies [6], and algorithms created through the use of time-lapse microscopy [7] were used to predict the destiny of day 3 embryos during in vitro culture. While ‘OMICS’ technologies, such as proteomics and metabolomics for non-invasive embryo developmental capacity assessment, are yet to be recommended for routine use [1], time-lapse microscopy has been introduced as a routine clinical practice and showed a capacity to predict the blastocyst formation with AUCs ranging from 0.6–0.8 across different studies [8–19]. Unfortunately, novel technologies inevitably require additional cost or equipment and the expense of technologies may limit their widespread use.

Although afflicted by subjectivity and limited efficacy, conventional embryo morphological assessment at fixed time point remained the standard of practice [20] in the era of ‘OMICS’ and time-lapse microscope. Since the early days of blastocyst culture, associations between day 3 morphology and blastocyst formation have been demonstrated. However, poor-looking day 3 embryos rejected by conventional embryo morphological assessment may also have a chance to develop into blastocysts and it is believed that the associations between morphology and blastocyst formation do not necessarily correlate with blastocyst viability. Nevertheless, data from studies predicting blastocyst formation using conventional morphological assessment and time-lapse microscopy in the same population [16, 18], showed that AUCs of conventional embryo morphological assessment for blastocyst formation were close to that of time-lapse microscopy. Especially, in the work of Petersen et al., which compared six time-lapse algorithms in the same study, only two algorithm surpassed an algorithm based on conventional Alpha/ESHRE consensus assessment in terms of predictive power [16]. Therefore, these data may suggest that the routine practice of laboratory remained a useful tool to predict blastocyst culture cancellation and provide meaningful clinical consultation, without additional cost or equipment. However, most of the previous studies focused on the assessment and selection of individual embryo and the performance of morphological based algorithms in predicting canceled blastocyst transfer cycle is less known. According to the previous studies [4, 5], there are still several other clinical and cycle based factors associated with blastocyst development besides the number and quality of day 3 embryos, and the morphology/ morphokinetic of day 3 embryos is also confounded by the cycle based factors they derived from [21]. The purpose of the study was to estimate the value of conventional embryo assessment until day 3 as tool to predict cycle based blastocyst-transfer cancellation rates.

In addition, contribution of cycle based factors to the predictive power was also evaluated by comparing the algorithms involving cycle based factors with those without.

## Materials and methods

### Study subjects

A retrospective analysis was performed on patients who underwent IVF/ICSI treatment in the Center for Reproduction Medicine of the affiliated Chenggong Hospital of Xiamen University, China, between January 2013 to December 2020. Institutional Review Board approval for this retrospective study was obtained from the Ethical Committee of the Medical College Xiamen University. Informed consent was not necessary, because the research was based on non-identifiable records as approved by the ethics committee.

The data from cycles in the period between January 2013 to December 2019 were obtained to create models to predict blastulation. The data from cycles in the period between January 2020 to December 2020 were obtained to validate the model. The inclusion criteria were the cycles accepted blastocyst culture and all parameters recorded precisely.

All patients were treated with conventional agonist or antagonist stimulation protocol in our center as previously described [22]. The initial and ongoing dosage was determined by patients’ age, antral follicle count (AFC), BMI, and ovarian response. When at least one follicle reached a mean diameter of 18 mm, An intramuscular injection of human chorionic gonadotropin (4000–6000 IU, hCG; Livzen, China) or a subcutaneous injection of recombinant human chorionic gonadotropin (250 μg, Ovidrel, Merck-Serono, Switzerland) was administrated for final triggering. Oocytes were retrieved under transvaginal ultrasound guidance 34–36 h after hCG injection.

### Embryo culture and assessment

Conventional IVF and ICSI protocol in our center were carried out [23]. After insemination, oocytes were cultured individually in preequilibrated Cleavage Medium (Cook) under mineral oil in traditional incubators (C200, Labotect) at 37℃, 6% CO2 and 5% O2 in a humidified atmosphere. In day3 morning, the culture media was switched to Blastocyst Medium (Cook) in the same culture condition. The culture system kept unchanged in the period of study.

Embryos were observed at the time according to Istanbul consensus [24]. Fertilization, early cleavage, the number and symmetry of blastomeres, fragmentation level on day 3 and blastocyst formation on day 5 and 6 were recorded.

### Statistical analysis

The blastocyst formation as the endpoint was defined as formation of viable blastocysts for either transfer for cryopreservation. Generalized estimating equations (GEE) were used to establish the morphology-based model. The features included in the model were early cleavage (with or without), the cell number on day 3 (2–3 cells, 4–6 cells, 7 cells, 8 cells, 9–11 cells, > 12 cells, and compact), fragmentation rate (continuous), and asymmetry (with or without). The grouping strategy for cell number on day 3 was based on the distribution of blastocyst formation (Figure S1).

To estimate whether including features regarding patient characteristics and cycle parameters improve the predicting power, we also establish models including 27 more features to establish additional models. The additional features were: female age, male age, GnRH analogues, insemination protocol, TESA/PESA, maternal height, maternal weight, maternal BMI, maternal basal FSH, maternal basal LH, maternal basal PRL, maternal basal E2, maternal basal T, basal AFC, gonadotropin dose, gonadotropin duration, HMG dose, HMG duration, starting dose, FSH on the day of stimulation, LH on the day of stimulation, E2 on the day of stimulation, E2 on the day of triggering, LH on the day of triggering, P on the day of triggering, oocyte yield, and maturation rate of oocytes in the cycle.

Two strategies were used to incorporate the features in the predicting models. First, a Least Absolute Shrinkage and Selection Operator (LASSO) model [25] was used for feature selection, and the resulting features along with morphological parameters were used to predict the blastocyst formation (LASSO model). Second, an Extreme Gradient Boosting (XGboost) algorithm [26] was used to establish gradient boosting trees with the features (XGboost model).

Predicting power of the models was quantified with the area under the receiver operating characteristic (ROC) curve with area under the curve (AUC). A 95% confidence interval (95% CI) was calculated for the AUC. A cutoff point for prediction was determined according to the maximum informedness (sensitivity + specificity-1) and the predictive value (PPV) and negative predictive value (NPV) of the given point were also calculated accordingly.

Because cancellation of blastocyst transfer was cycle based, we also attempted to evaluate the clinical usefulness of the blastocyst formation prediction of individual embryos in a given cycle. Models were used to calculate the predicted number of blastocysts in cycles. The predicted number of blastocyst correlated to the observed number of the blastocyst with Spearman‘s rank correlation and mean absolute difference between the prediction and observation was calculated.

The cumulative probability of predicted blastocyst formation of individual embryos in a cycle was used to predict whether the cycle have blastocyst for transfer. The cumulative probability was defined as follows. Cumulative probability = 1-∏(1-individulal embryo blastocyst formation rate).

The predicting power was compared to a cycle-based model based on XGboost with 29 features. The features included the aforementioned patient characteristics and cycle parameters, as well as the number of good quality embryos and whether all cleavages were subjected to blastocyst culture in cycles.

Calibration curves were used to report clinical agreement between model predictions and observed outcomes in the large. A calibration curve was plotted by comparing the relationship between model values and observed rates, grouped by deciles of model values. When the predicted number of blastocysts were used for prediction, a logistical transfer was used in order to obtain a linear relationship.

The calibration slope was used to evaluate the spread of the estimated rates with a target value of 1. A slope < 1 suggests that the prediction was too extreme and a slope > 1 suggests the opposite. The calibration intercept with a target value of 0, was an assessment of calibration-in-the-large. The negative intercept suggested overestimation, whereas positive intercept suggest underestimation.

All analyses were performed using R Statistical Software (v4.1.2, R Core Team 2021).

## Result

Training data included 13,674 cycles. The median of maternal age is 30[28-33]. 3010(23.1%) cycles accepted ICSI and 10,038(76.9%) accepted IVF treatment. A total of 96,378 embryos were cultured for blastulation. Early cleavage occurred in 42,669(44.3%) embryos. In the end, 55,323(57.4%) embryos developed to blastocysts. Another 1956 cycles were included to validate the model. The median of maternal age is 31[29-34]. 506(25.9%) cycles accepted ICSI and 1450(74.1%) accepted IVF treatment. A total of 11,770 embryos were cultured for blastulation. Early cleavage occurred in 5961(50.6%) embryos. In the end, 7024(59.7%) embryos developed to blastocysts (Table 1 and 2).

**Table 1:** Cycle characteristics of patients

Variable	Training set	Validation set
Cycles, n	13,657	1956
Female age, year	30 [28-33]	31 [29-34]
Male age, year	32 [29-35]	32 [30–36]
GnRH analogues		
Agonist	12,118(88.7)	1605(82.1)
Non-agonist	1539(11.3)	351(17.9)
Insemination protocol		
IVF	9948(72.8)	1450(74.1)
ICSI	3709(27.2)	506(25.9)
TESA/PESA + ICSI	643(4.7)	72(3.7)
Female height, cm	158 [155–162]	158 [155–162]
Female weight, kg	53 [48–57]	53 [49–57]
Female BMI, kg/m2	21 [19.4–22.6]	21.33 [19.78–22.77]
Female basal FSH, IU/l	6.71 [5.76–7.88]	7.68 [6.4325–9.17]
Female basal LH, IU/l	4.41 [3.29–5.9]	4.55 [3.4–6.25]
Female basal PRL, ng/ml	13.83 [10.03–19.11]	14.74 [10.8725–20.6175]
Female basal E2, pg/ml	41 [30–55]	42 [31–57]
Female basal T, ng/ml	0.42 [0.3–0.56]	0.42 [0.3–0.55]
Basal AFC	11 [8-15]	10 [7-15]
Gonadotropin dose, IU	2250 [1800–2700]	2250 [1800–2700]
Gonadotropin duration, IU	12 [10-13]	12 [10-13]
HMG dose, IU	1987.5 [937.5–2475]	1950 [675–2550]
HMG duration, IU	11 [8-12]	11 [5-13]
Starting dose, IU	225 [150–225]	187.5 [150–225]
FSH on the day of stimulation, IU/l	2.34 [1.59–3.58]	2.74 [1.88–4.64]
LH on the day of stimulation, IU/l	0.81 [0.58–1.17]	0.79 [0.55–1.2875]
E2 on the day of stimulation, pg/ml	20 [12-30]	23 [14-35]
E2 on the day of triggering, pg/ml	3845 [2390–5198]	3231.5 [1850.75–4754.5]
LH on the day of triggering, IU/l	0.69 [0.41–1.13]	0.7 [0.39–1.29]
P on the day of triggering, ng/ml	1.03 [0.71–1.46]	0.86 [0.6–1.2]
Oocyte yield	11 [8-16]	10 [6-13]
Maturation rate of oocytes,%	92.8 [84.6–100]	92.85 [83.33–100]
Embryos subjected to blastocyst culture	6 [4-9]	5 [3-8]
Good quality embryos	5 [3-7]	4 [2-6]
Cycles with no blastocyst formed, %	1008(7.4)	213(10.9)
Blasocyst formed, per cycle	3 [2-6]	3 [1-5]
Blastulation rate, per cycle	58.33 [37.5–75]	61.54 [38.46–81.82]

**Table 2:** Characteristics of embryos subjected to blastocyst culture

	Training set	Validation set
Total embryos cultured	96,378	11,770
Fragmentation, %	0[0–5]	0[0–5]
Cell number on day 3 (%)		
8 cells	32,992(34.2)	4240(36.0)
2–3 cells	3678(3.8)	318(2.7)
4–6 cells	23,278(24.2)	2844(24.2)
7 cells	16,147(16.8)	1873(15.9)
9–11 cells	13,912(14.4)	1758(14.9)
12–15 cells	2814(2.9)	501(4.3)
compact	3557(3.7)	236(2.0)
Symmetry (%)		
uneven	26,956(28)	2000(17)
even	69,422(72)	9770(83)
Early cleavage (%)		
yes	42,669(44.3)	5961(50.6)
no	53,709(55.7)	5809(49.4)
Total blastocyst formed (%)	55,323(57.4)	7024(59.7)

Based on early cleavage and routine observed morphological parameters (cell number, fragmentation, and symmetry), we established a predicting model with GEE. The coefficients and interception was shown in Supplementary Table 1 (Table S1). The GEE model predicted blastocyst formation with an AUC of 0.771(95%CI: 0.768–0.774) in the training set and 0.779(95%CI: 0.77–0.787) in the validation set. A cutoff of 0.51 was determined according to the maximum informedness. The accuracy of prediction according to the cutoff was 74.7%(95%CI: 73.9%-75.5%) in the validation set. Similarly, LASSO regression model and XGboost model based on the combination of cycle characteristics and embryo morphology yielded similar predicting power with AUCs of 0.78(95%CI: 0.771–0.789) and 0.754(95%CI: 0.745–0.763) in validation set, respectively. There was no significant difference in terms of predicting power demonstrated with AUCs among different model (Table 3). The AUC curves and calibration curves were also comparable (Figure S2).

**Table 3:** Discrimination of different models in predicting blastocyst formation

	Training set							Validation set			
Model	AUC (95%CI)	Cutoff	Sensitivity(95%CI)	Specificity (95%CI)	PPV(95%CI)	NPV(95%CI)	Accuracy(95%CI)	AUC (95%CI)	PPV(95%CI)	NPV(95%CI)	Accuracy(95%CI)
GEE^a^	0.771(0.768–0.774)	0.51	0.804(0.801–0.808)	0.625(0.62–0.629)	0.743(0.739–0.746)	0.703(0.698–0.708)	0.728(0.725–0.731)	0.779(0.77–0.787)	0.761(0.751–0.77)	0.721(0.707–0.735)	0.747(0.739–0.755)
LASSO^b^	0.775(0.772–0.778)	0.55	0.793(0.789–0.796)	0.636(0.631–0.64)	0.746(0.742–0.749)	0.695(0.69–0.699)	0.726(0.723–0.729)	0.78(0.771–0.789)	0.77(0.761–0.78)	0.699(0.686–0.713)	0.744(0.736–0.752)
Xgboost^b^	0.783(0.78–0.785)	0.57	0.8(0.796–0.803)	0.635(0.63–0.639)	0.747(0.743–0.75)	0.701(0.697–0.706)	0.729(0.726–0.732)	0.754(0.745–0.763)	0.773(0.763–0.783)	0.645(0.632–0.659)	0.719(0.711–0.727)

^a^model includes embryo morphology only

^b^model includes following features in combination with embryo morphology: female age, male age, GnRH analogues, insemination protocol, TESA/PESA, maternal height, maternal weight, maternal BMI, maternal basal FSH, maternal basal LH, maternal basal PRL, maternal basal E2, maternal basal T, basal AFC, gonadotropin dose, gonadotropin duration, HMG dose, HMG duration, starting dose, FSH on the day of stimulation, LH on the day of stimulation, E2 on the day of stimulation, E2 on the day of triggering, LH on the day of triggering, P on the day of triggering, oocyte yield, and maturation rate of oocytes in the cycle

We further explored the discrimination of GEE model with the given cutoff value for blastocyst formation in different subgroup of patients (Table 4). The predicting power in terms of AUCs were similar in the large and denoted a fair performance. However, the discrimination power appeared to be lower in aged patients and patients with fewer oocyte yield.

**Table 4:** Discrimination of morphology-only model in predicting blastocyst formation in different patient subgroup

	Training set						Validation set				
Subgroup	n	Prevalence(95%CI)	AUC(95%CI)	PPV(95%CI)	NPV(95%CI)	Accuracy(95%CI)	n	Prevalence(95%CI)	AUC(95%CI)	PPV(95%CI)	NPV(95%CI)
Insemination											
IVF	70,073	0.592(0.588–0.596)	0.771(0.767–0.775)	0.753(0.749–0.757)	0.697(0.691–0.702)	0.733(0.73–0.736)	8615	0.615(0.605–0.625)	0.794(0.784–0.804)	0.769(0.759–0.78)	0.722(0.706–0.739)
ICSI	26,305	0.526(0.52–0.532)	0.767(0.761–0.772)	0.712(0.705–0.719)	0.717(0.709–0.725)	0.714(0.709–0.72)	3155	0.546(0.529–0.564)	0.781(0.765–0.797)	0.733(0.713–0.753)	0.719(0.694–0.743)
Female age											
age < 35	82,599	0.581(0.578–0.584)	0.775(0.772–0.779)	0.75(0.747–0.754)	0.698(0.693–0.703)	0.731(0.728–0.734)	9603	0.604(0.594–0.614)	0.798(0.789–0.807)	0.771(0.76–0.781)	0.717(0.702–0.733)
age≧35	13,779	0.532(0.524–0.541)	0.749(0.741–0.758)	0.697(0.688–0.707)	0.732(0.72–0.744)	0.711(0.703–0.718)	2167	0.565(0.544–0.586)	0.767(0.747–0.787)	0.716(0.693–0.739)	0.741(0.709–0.773)
Ovarian response											
oocyte≦4	1677	0.592(0.568–0.615)	0.741(0.716–0.765)	0.741(0.714–0.767)	0.66(0.623–0.697)	0.711(0.689–0.732)	434	0.585(0.539–0.632)	0.752(0.706–0.798)	0.717(0.665–0.77)	0.662(0.587–0.738)
oocyte > 4	94,701	0.574(0.571–0.577)	0.772(0.769–0.775)	0.743(0.739–0.746)	0.704(0.699–0.709)	0.728(0.725–0.731)	11,336	0.597(0.588–0.606)	0.794(0.785–0.802)	0.762(0.753–0.772)	0.724(0.71–0.738)

In clinical practice, whether a blastocyst transfer cycle is canceled may be determined by the availability of all embryos subjected to blastocyst formation in the cycle. To mimic the scenario, we further generated a per-cycle blastocysts prediction based on the models. The predicted number of blastocyst per cycle was simply the sum of individual embryo prediction. For per-cycle blastocyst yield, the predicted number of blastocysts using morphological parameters alone strongly correlated with observed blastocyst number (*r* = 0.897, *P* < 0.0001) with a mean absolute error of 0.95 (95%CI: 0.92–0.99).

**Fig. 1:**
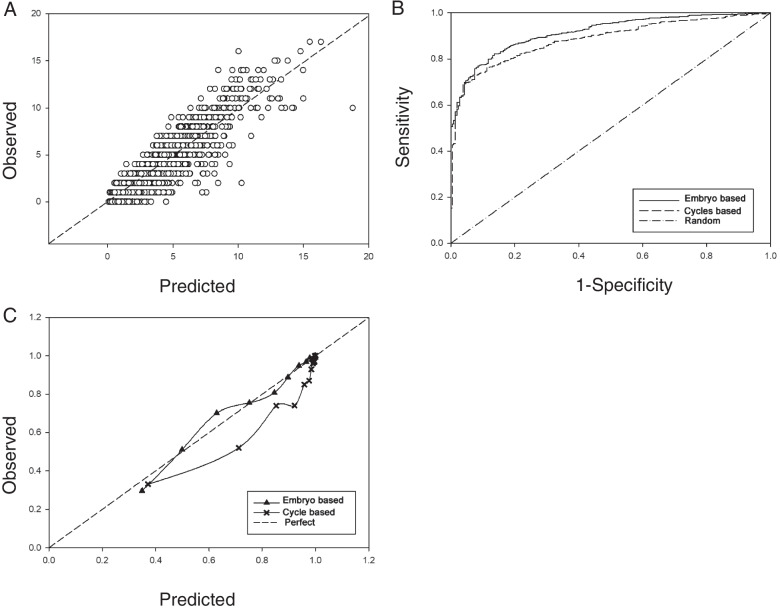
Performance of embryo based and cycle based model in predicting cycle with blastocyst. **A** Scatter plot indicating correlation between observed blastocyst number and predicted blastocyst number based on embryo morphology. **B** ROC curves of embryo based model and cycle based model to predict cycle with blastocyst. **C** Calibration curves linking predicted probability and observed proportion of cycles with blastocyst according to embryo based and cycle based model

The predicted number of blastocysts was also used to predict chance of blastocyst transfer with an AUC of 0.926((95%CI: 0.911–0.94). The predicting power of the predicted number of blastocysts for blastocyst transfer cancel surpassed an XGBoost model based on 29 features (AUC 0.885, 95%CI: 0.867- 0.903). Figure 1 demonstrated the AUCs and calibration curves of both models. The cycles based model appeared to be overestimate the chance of blastocyst transfer (slope = 1.01, intercept = -0.009) while the predicted number of blastocyst made a prediction closer to observed probability (slope = 1.15, intercept = -0.185).

In Table 5, the prediction of blastocyst transfer was stratified according to patient subgroup. The predictive power in terms of AUC of ROC curves was not significantly differ in patients older than 34 years in comparison with the unselected population. On the other hand, the patients with no good quality embryos and the patients with partial embryos cultured suffered a decreased AUC. However, the AUC in the patients with no good quality embryos still suggested a moderate discriminability with a value of 0.74 (95% CI: 0.68–0.79).

**Table 5:** Discrimination of embryo morphology to predict the cycle with blastocyst in different patient subgroup

Subgroup	n	Prevalence (95%CI)	AUC (95%CI)	Threshold	Sensitivity (95%CI)	Specificity (95%CI)	PPV (95%CI)	NPV (95%CI)	Accuracy (95%CI)
Overall	1956	0.89(0.88–0.9)	0.93(0.92–0.94)	predicted embryo≧1	0.92(0.9–0.93)	0.7(0.64–0.76)	0.96(0.95–0.97)	0.5(0.45–0.56)	0.89(0.88–0.91)
				cumulative *p*≧0.9	0.85(0.83–0.87)	0.88(0.83–0.92)	0.98(0.98–0.99)	0.41(0.37–0.46)	0.85(0.84–0.87)
				cumulative *p*≧0.8	0.91(0.9–0.92)	0.74(0.68–0.8)	0.97(0.96–0.97)	0.5(0.44–0.56)	0.89(0.88–0.9)
				cumulative *p*≧0.7	0.95(0.94–0.96)	0.6(0.53–0.66)	0.95(0.94–0.96)	0.59(0.52–0.65)	0.91(0.9–0.92)
				cumulative *p*≧0.6	0.96(0.95–0.97)	0.51(0.44–0.58)	0.94(0.93–0.95)	0.62(0.55–0.69)	0.91(0.9–0.93)
				cumulative *p*≧0.5	0.98(0.97–0.98)	0.39(0.33–0.46)	0.93(0.92–0.94)	0.66(0.58–0.74)	0.91(0.9–0.92)
Embryo cultured < 4	624	0.71(0.67–0.74)	0.81(0.78–0.85)	predicted embryo≧1	0.68(0.63–0.72)	0.77(0.71–0.83)	0.88(0.84–0.91)	0.5(0.44–0.55)	0.7(0.67–0.74)
				cumulative *p*≧0.9	0.5(0.46–0.55)	0.92(0.88–0.96)	0.94(0.91–0.97)	0.44(0.39–0.48)	0.63(0.59–0.66)
				cumulative *p*≧0.8	0.68(0.64–0.72)	0.79(0.73–0.85)	0.89(0.85–0.92)	0.51(0.45–0.56)	0.71(0.68–0.75)
				cumulative *p*≧0.7	0.81(0.77–0.84)	0.65(0.58–0.72)	0.85(0.81–0.88)	0.58(0.52–0.65)	0.76(0.73–0.79)
				cumulative *p*≧0.6	0.85(0.82–0.89)	0.56(0.49–0.63)	0.82(0.79–0.86)	0.62(0.54–0.69)	0.77(0.74–0.8)
				cumulative *p*≧0.5	0.91(0.88–0.93)	0.43(0.36–0.5)	0.79(0.76–0.83)	0.66(0.57–0.74)	0.77(0.73–0.8)
Female age≧35	461	0.82(0.78–0.85)	0.91(0.88–0.94)	predicted embryo≧1	0.89(0.86–0.92)	0.72(0.62–0.81)	0.93(0.91–0.96)	0.6(0.5–0.69)	0.86(0.83–0.89)
				cumulative *p*≧0.9	0.82(0.79–0.86)	0.87(0.8–0.94)	0.97(0.95–0.99)	0.53(0.45–0.61)	0.83(0.8–0.87)
				cumulative *p*≧0.8	0.89(0.86–0.92)	0.73(0.63–0.82)	0.94(0.91–0.96)	0.6(0.51–0.7)	0.86(0.83–0.89)
				cumulative *p*≧0.7	0.94(0.92–0.97)	0.58(0.47–0.68)	0.91(0.88–0.94)	0.7(0.59–0.81)	0.88(0.85–0.91)
				cumulative *p*≧0.6	0.95(0.93–0.97)	0.44(0.33–0.54)	0.88(0.85–0.91)	0.67(0.55–0.8)	0.86(0.82–0.89)
				cumulative *p*≧0.5	0.98(0.97–1)	0.33(0.23–0.43)	0.87(0.83–0.9)	0.82(0.7–0.95)	0.86(0.83–0.89)
Without good embryo	279	0.51(0.45–0.57)	0.74(0.68–0.79)	predicted embryo≧1	0.43(0.35–0.51)	0.85(0.78–0.91)	0.75(0.65–0.84)	0.59(0.52–0.66)	0.63(0.58–0.69)
				cumulative *p*≧0.9	0.19(0.12–0.25)	0.98(0.95–1)	0.9(0.79–1.01)	0.53(0.47–0.6)	0.57(0.52–0.63)
				cumulative *p*≧0.8	0.36(0.28–0.44)	0.91(0.86–0.96)	0.81(0.72–0.91)	0.58(0.51–0.64)	0.63(0.57–0.69)
				cumulative *p*≧0.7	0.53(0.45–0.61)	0.84(0.78–0.9)	0.78(0.69–0.86)	0.63(0.56–0.7)	0.68(0.63–0.74)
				cumulative *p*≧0.6	0.59(0.51–0.67)	0.77(0.7–0.84)	0.73(0.65–0.81)	0.64(0.57–0.71)	0.68(0.62–0.73)
				cumulative *p*≧0.5	0.73(0.65–0.8)	0.62(0.54–0.7)	0.67(0.59–0.74)	0.68(0.6–0.77)	0.67(0.62–0.73)
Partial embryo cultured	863	0.81(0.78–0.84)	0.87(0.84–0.9)	predicted embryo≧1	0.84(0.81–0.87)	0.72(0.65–0.78)	0.93(0.91–0.95)	0.51(0.45–0.58)	0.81(0.79–0.84)
				cumulatvie *p*≧0.9	0.68(0.65–0.72)	0.9(0.85–0.94)	0.97(0.95–0.98)	0.4(0.35–0.45)	0.72(0.69–0.75)
				cumulatvie *p*≧0.8	0.82(0.79–0.85)	0.78(0.72–0.84)	0.94(0.92–0.96)	0.51(0.45–0.57)	0.81(0.79–0.84)
				cumulatvie *p*≧0.7	0.9(0.87–0.92)	0.62(0.55–0.7)	0.91(0.89–0.93)	0.59(0.51–0.66)	0.84(0.82–0.87)
				cumulatvie *p*≧0.6	0.92(0.9–0.94)	0.53(0.46–0.61)	0.89(0.87–0.92)	0.61(0.53–0.69)	0.85(0.82–0.87)
				cumulatvie *p*≧0.5	0.95(0.94–0.97)	0.42(0.35–0.5)	0.87(0.85–0.9)	0.67(0.58–0.76)	0.85(0.83–0.87)

## Discussion

Although challenged by novel technologies for embryo assessment, the conventional static morphological assessment is still widespread used with established consensus of practice [24, 27], generating large amount of datasets within the past decades. A feasible clinical prediction model based on these datasets may benefit from the large sample size and be easily incorporated in the routine procedures without additional cost. In the present study, we demonstrated the predictive values of conventional static morphological assessment for blastocyst formation and provided a simplified predicting algorithm to predicted canceled blastocyst transfer cycles with a moderate predictive power. In addition, by comparing with models including cycle based parameters, our data also suggested that increase the complexity of the model by taking parameters other than the embryo themselves may not significantly improved the predictive power.

Since the early days of blastocyst culture, many previous studies have investigated the association between conventional morphology assessment and the rates of blastocyst formation [28–30]. However, only a few studies quantitatively evaluated the predictive power [16, 18]. Basile et al. evaluated the predictive power for blastocyst formation of morphological criteria defined by the Spanish Association of Embryologists (ASEBIR), showing an AUC of 0.717 (CI95%: 0.703–0.732), which is close to the AUC derived from Eeva time lapse system in the same population. In a classic paper comparing multiple blastocyst formation algorithms, the algorithm based on conventional Istanbul consensus showed an AUC of 0.700 (CI95%: 0.687–0.714), surpassing several time-lapse based algorithms in the same population. Both studies focused on the comparison of different algorithms and the authors may aim at “giving all investigated algorithms equal frames”. Therefore, both time-lapse and conventional morphology results were given as classifications or score groups and the contribution of each conventional morphology parameters were not demonstrated. In addition, the work of Petersen et al. used only the timings part of Istanbul consensus. Comparing with the previous studies, our algorithms based on data-driven models using a full set of conventional morphology parameters. Including conventional morphology parameters rather than pre-established classifications may provide more information and therefore increase the predictive power.

The time-lapse microscopy, which generates thousands of images during the in vitro culture of an embryo, provides far more information than conventional static observation. Theoretically, this advantage may make it a superior morphology tool to predict the fate of a cultured embryo. However, several earlier studies did not demonstrate satisfying predictive power for blastocyst formation, with AUCs ranging from 0.6 to 0.7. More recently, the time-lapse algorithm developed by Motato et al., predicted blastocyst formation with an AUC value 0.849 (95% CI: 0.835–0.854) [17]. This method, however, requires a culture duration up to 96 h, which may resulted in a delayed decision making. A recent study integrates deep learning algorithms to the time-lapse system, and the predictive power in terms of AUC reaches 0.82 [8]. In comparison the historical performance of time-lapse system in predicting blastocyst formation, the conventional static observation of the old era yields acceptable predictive power and only requires limited resource.

Most of the previous morphological studies focused on tracking the destiny of an individual embryo, aiming to select the most competent embryo. On the other hand, whether blastocyst culture yields viable blastocysts for transfer also relates to the decision making. It has been proposed that four good embryos on day 3 may reassure that the patients will benefit from blastocyst transfer [31]. However, the performance of a day 3 morphology based algorithm to predict a canceled blastocyst transfer cycle due to failed blastocyst formation yet to be determined. A few studies used cycle based characteristics, such as maternal age, oocyte yield and the number of good-quality embryos, to predict the probability of blastocyst transfer cancellation [4, 5]. Dessolle et al. established a cycle-based model with multivariate logistical regression and showed a AUC of 0.75 (95% CI: 0.73–0.77) for predicting canceled cycles [4]. More recently, a model using multiple classification algorithms predicted cycle based blastocyst formation with an AUC of 0.922 [5]. Both algorithms require the patient characteristics in combination with embryo quality on day 3. Our data suggested that the cumulative probability of morphological assessment based prediction alone also yields a notable prediction power. Independent of cycle based characteristics, the prediction may be more flexible as it is also applicable to the cycles where only a part of embryos is subjected to blastocyst culture. Interestingly, both our cycle based model and model of Dessolle et al. suggested a tendency of overestimation according to the calibration plots, although different statistic methods and populations were involved. It may suggest a potential intrinsic feature of cycle based prediction.

Prevalence of blastocyst formation in different population may be another issue should be considered beyond discriminability when attempt is made to predict the chance of embryo transfer in a given cycle. The rates of blastocyst formation vary widely among patients, ranging from 0% to almost 100% [1]. In unselected population, the chance to have at least one blastocyst to transfer in a cycle may be rather high. For instance, Dessolle et al. observed that the percentage of cycles with blastocyst transfer was about 79% in the study cohort [4]. We also observed a cycle based blastocyst formation rates approximating 90% in the overall population. With a high prevalence, a naive guessing by always predicting ‘yes’ could still yield high accuracy. On the other hand, however, patients with low embryo yield or advanced age may suffer a higher chance of blastocyst culture failure [2, 3], and need a more detailed consults before making decision. Therefore, we sought to evaluate the discriminability of the model in different subgroup of patients, which may represent different scenarios, and the reasonable AUCs were observed. Notably, a remarkable decrease in blastocyst formation rate was observed among the patients who had no good-quality embryo for blastocyst culture, while a moderate discriminability was observed in the ROC curve. The results suggested that conventional morphology observation remains a useful consulting tool, even no good quality embryo was scored.

It is known that in vitro development of embryos is associated with patient- and treatment-related factors [21]. Beyond the morphology/morphokinetic characteristics, the patient- and treatment-related factors may also affect intrinsic characteristics, such as aneuploidy or metabolism [32, 33]. These characteristics may not be necessarily associated with the appearance of the embryos. Therefore, adding factors such as age may increase the information available for the prediction. To test this hypothesis, we compared the multivariate model including only morphological parameters with models constructed with LASSO regression or XGboost including both morphological parameters and patient/treatment related factors. Although the patient/treatment related factors were significantly associated with blastulation, the LASSO/XGboost models did not significantly surpass the simple multivariate model in terms of predictive power. Well-known prognosis factors for blastocyst formation, such as AMH and maternal age also affected the quantity and morphology of day 3 embryos [34, 35]. The existence of mediation effects, where day 3 embryo quality serve as a mediator, may partially explain why including more patient/treatment related factors may not further improve the performance of the model in the study.

The study is fortified by a large sample size, which may provide a narrow confidence interval for coefficients and reduce the uncertainty of the performance. In addition, we also calibrate the model in several different clinical scenarios, including advanced maternal age, few embryos for culture and blastocyst culture for surplus embryos. The study also suffered from several drawbacks, including retrospective design and subjectivity of methodology. Because the study is single-centered, we could not test the performance of the model in other culture system. Nevertheless, the study may encourage the establishment of predicting model based on existing large datasets in other culture system.

## Conclusion

In the present study, data suggested that conventional morphology remained a useful tool to predict blastocyst formation and blastocyst transfer cancellation. Meaningful consult on blastocyst culture could be made on day 3 morning based on a combination of morphological parameters, even though no good quality embryo was obtained at the time.

## Supplementary Information

**Additional file 1:** **Figures S1.** Distribution of blastulation rate among different day 3 embryos with different cell number in the training data. Error bar indicates 95% CI of the rates.

**Additional file 2:** **Figure S2.** Performance of models in predicting blastocyst formation. (A) ROC curves for models (B) Calibration plot linking predicted probabilities to observed rates rates.

**Additional file 3:** **Table S1.** Model coefficients of GEE and LASSO model to predict blastocyst formation.

## Data Availability

Not applicable.
