# Supplementary figures and images for: Does conventional morphological evaluation still play a role in predicting blastocyst formation?
Source: Reprod Biol Endocrinol. 2022 Apr 19;20:68. doi: 10.1186/s12958-022-00945-y (PMC9016972; doi:10.1186/s12958-022-00945-y)

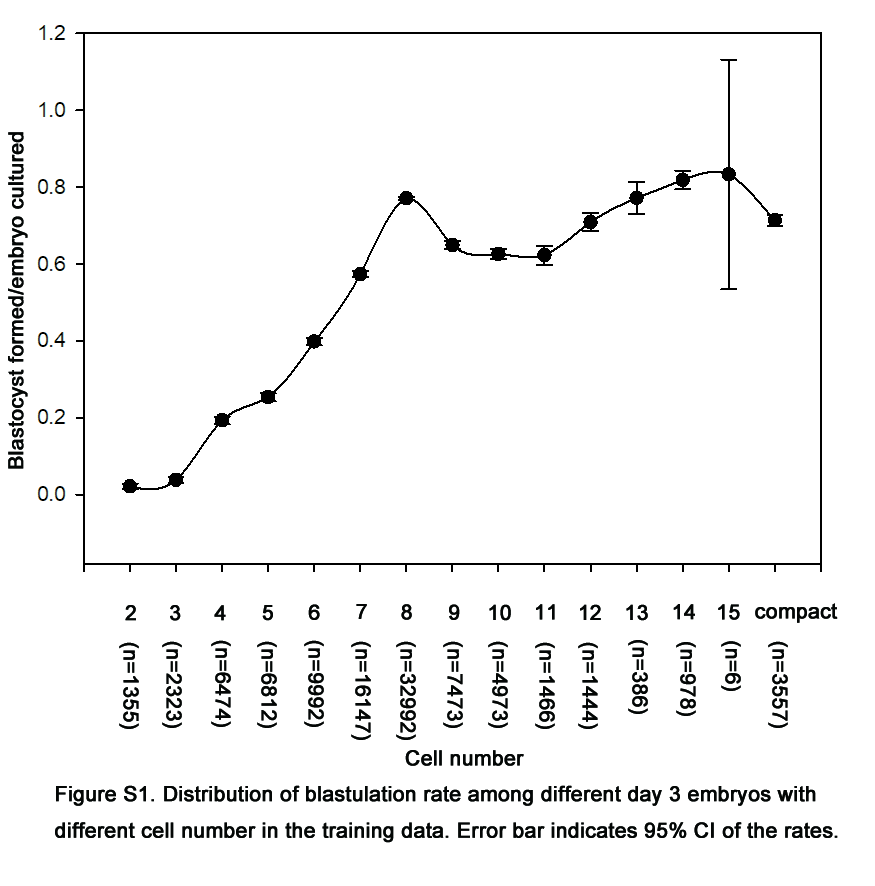

Supplement: Supplementary file 1 — Additional file 1: Figures S1. Distribution of blastulation rate among different day 3 embryos with different cell number in the training data. Error bar indicates 95% CI of the rates. [file 12958_2022_945_MOESM1_ESM.tif]

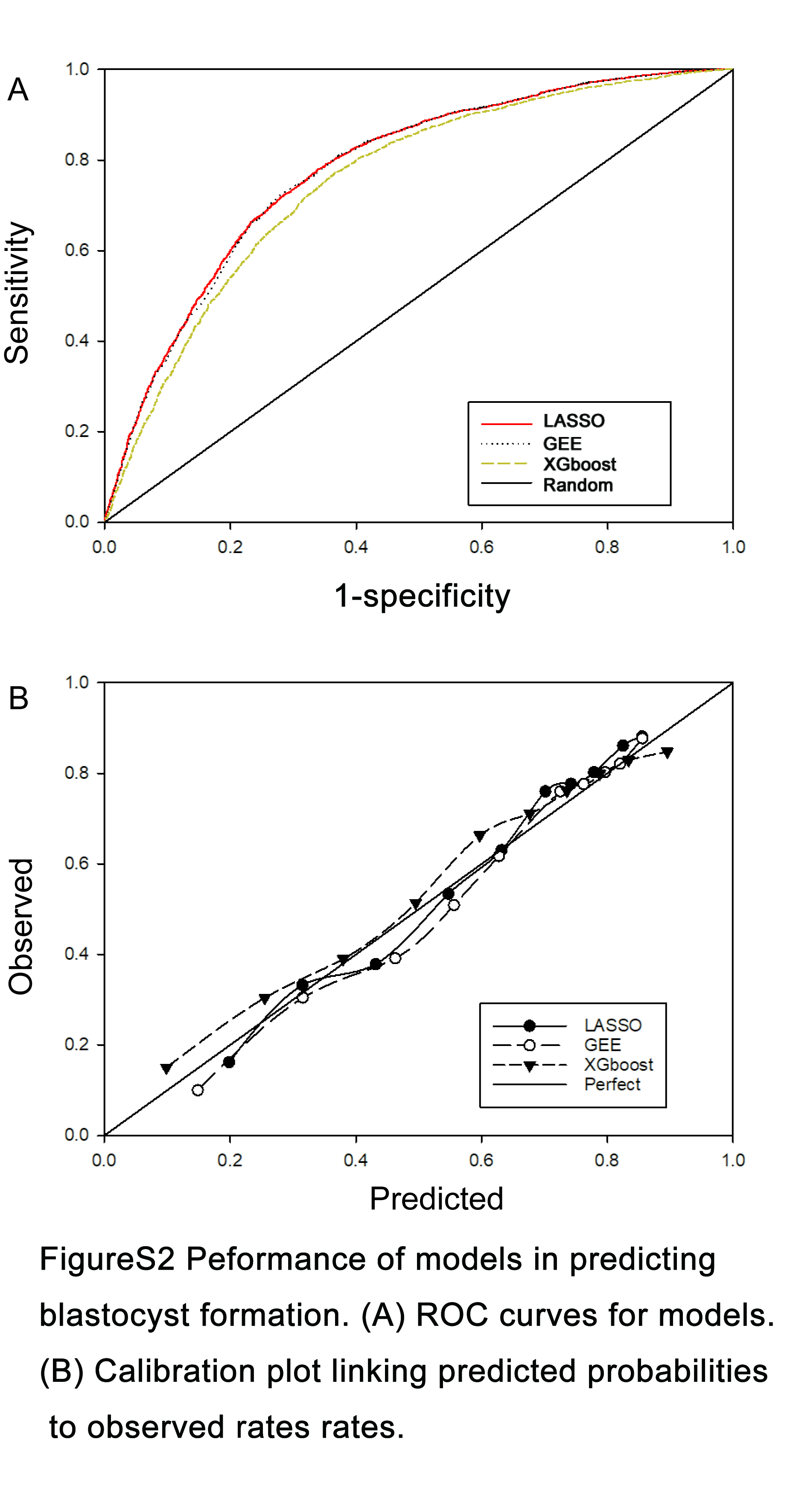

Supplement: Supplementary file 2 — Additional file 2: Figure S2. Performance of models in predicting blastocyst formation. (A) ROC curves for models (B) Calibration plot linking predicted probabilities to observed rates rates. [file 12958_2022_945_MOESM2_ESM.tif]
