# Supplementary material for: Does conventional morphological evaluation still play a role in predicting blastocyst formation?
Source: Reprod Biol Endocrinol. 2022 Apr 19;20:68. doi: 10.1186/s12958-022-00945-y (PMC9016972; doi:10.1186/s12958-022-00945-y)
Supplement: Supplementary file 3 — Additional file 3: Table S1. Model coefficients of GEE and LASSO model to predict blastocyst formation. [file 12958_2022_945_MOESM3_ESM.docx]

Table S1 Model coefficients of GEE and LASSO model to predict blastocyst formation

| Model coefficient | GEE | LASSO |
| --- | --- | --- |
| (Intercept) | 1.101 | 1.08 |
| Early cleavage |  |  |
| Cell number on day 3 | 0.682 | 0.68 |
| 8 cells | ref |  |
| 2-3 cells | -4.327 | -4.20 |
| 4-6 cells | -1.768 | -1.74 |
| 7 cells | -0.665 | -0.64 |
| 9-11 cells | -0.583 | -0.55 |
| 12-15 cells | -0.303 | -0.26 |
| compact | -0.456 | -0.41 |
| Day 3 fragmentation, perecntage | -0.053 | -0.05 |
| symmetry |  |  |
| even | ref | ref |
| uneven | -0.518 | -0.5104 |
| Female age, per year | - | -0.0243 |
| Male age, per year | - | reduced |
| Agonist |  |  |
| no | - |  |
| yes | - | 0.0108 |
| ICSI |  |  |
| no | - |  |
| yes | - | -0.1215 |
|  |  |  |
| PESA/TESA | - | 0.0193 |
| Female height, per cm | - | -0.0012 |
| Female weight, per kg | - | 0.0026 |
| Female BMI, per unit | - | reduced |
| Basal FSH, per unit | - | -0.0004 |
| Basal LH | - | -0.0002 |
| Basal PRL | - | reduced |
| Basal E2 | - | 0.0000 |
| Basal T | - | -0.0009 |
| Basal AFC | - | 0.0034 |
| Gonadotropin dose, per IU | - | -0.0001 |
| Gonadotropin duration, per day | - | -0.0015 |
| HMG dose, per unit | - | reduced |
| HMG duration per day | - | 0.0009 |
| Starting dose, per IU | - | reduced |
| FSH on the day of stimulation, per IU/l | - | -0.0011 |
| LH on the day of stimulation, per IU/l | - | -0.0001 |
| E2 on the day of stimulation, per pg/ml | - | 0.0020 |
| E2 on the day of triggering, per pg/ml | - | 0.0000 |
| LH on the day of triggering, per IU/l | - | reduced |
| P on the day of triggering, per ng/ml | - | -0.0276 |
| Oocyte yield | - | -0.0039 |
| Maturation rate of oocytes ,% | - | 0.0004 |
